# Why live recording sounds better: a case study of Schumann's *Träumerei*

**DOI:** 10.3389/fpsyg.2014.01564

**Published:** 2015-01-09

**Authors:** Haruka Shoda, Mayumi Adachi

**Affiliations:** ^1^Department of Culture and Information Science, Doshisha UniversityKyotanabe, Japan; ^2^Japan Society for the Promotion of ScienceTokyo, Japan; ^3^Department of Psychology, Hokkaido UniversitySapporo, Japan

**Keywords:** music, live recording, social facilitation, listeners' evaluation, acoustical analysis, functional principal components analysis, multi-group path analysis

## Abstract

We explore the concept that artists perform best in front of an audience. The negative effects of performance anxiety are much better known than their related cousin on the other shoulder: the positive effects of “social facilitation.” The present study, however, reveals a listener's preference for performances recorded in front of an audience. In Study 1, we prepared two types of recordings of *Träumerei* performed by 13 pianists: recordings in front of an audience and those with no audience. According to the evaluation by 153 listeners, the recordings performed in front of an audience sounded better, suggesting that the presence of an audience enhanced or facilitated the performance. In Study 2, we analyzed pianists' durational and dynamic expressions. According to the functional principal components analyses, we found that the expression of “*Träumerei*” consisted of three components: the overall quantity, the cross-sectional contrast between the final and the remaining sections, and the control of the expressive variability. Pianists' expressions were targeted more to the “average” of the cross-sectional variation in the audience-present than in the audience-absent recordings. In Study 3, we explored a model that explained listeners' responses induced by pianists' acoustical expressions, using path analyses. The final model indicated that the cross-sectional variation of the duration and that of the dynamics determined listeners' evaluations of the quality and the emotionally moving experience, respectively. In line with human's preferences for commonality, the more “average” the durational expressions were in live recording, the better the listeners' evaluations were regardless of their musical experiences. Only the well-experienced listeners (at least 16 years of musical training) were moved more by the “deviated” dynamic expressions in live recording, suggesting a link between the experienced listener's emotional experience and the unique dynamics in music.

## Introduction

Music has been played to an audience for millennia. Studio recording of music is a relatively new phenomena. Some performers prefer live to studio recordings because of their serendipitous experiences: “The live recording promises the excitement of a unique event and the moments of tension and inspiration that can only occur during a complete performance in front of an audience” (Badal, 1996, p. 10). Listeners who prefer live to studio recordings also believe that they can feel performers' passions from the live recordings (Badal, 1996). Do recordings made in front of an audience actually sound better for listeners than those made alone? If yes, how do these recordings differ acoustically, and how exactly do such differences determine listeners' experiences of the two types of recording? We explored these ideas in the present study.

Researchers in performance science have explored factors determining performers' expressions. For example, Hargreaves et al. (2005) categorized them qualitatively into three groups: “music” (e.g., genres, idioms, complexity, styles, familiarity), “performer” (e.g., instrumental/vocal, solo/group, gender, age, personality, internal state), and “situations and contexts” (e.g., social and cultural context, presence/absence of others). Previous studies have empirically confirmed that styles (Baroque, Romantic, Modern, e.g., Shaffer and Todd, 1994), performers' age (e.g., children, Adachi et al., 2004), and cultural contexts (e.g., Clayton, 2005) actually influence performers' expressions of performance parameters (e.g., tempo, dynamics, timbre). In the present study, we quantitatively explored effects of an audience—“situations and contexts” in Hargreaves et al. (2005)—on the quality of performers' expressions.

The literature has often claimed the presence of an audience to be a “stressor” for performers, which can paralyze their mental and physical conditions. In a competition (Yoshie et al., 2009a,b), for example, performers are likely to experience cognitive anxiety (e.g., “I am concerned about choking under pressure”) and somatic one (e.g., “My heart is racing”), accompanied with stress-related physiological responses such as accelerated heart rate, increased electromyographic activity, and more sweat (e.g., Yoshie et al., 2009a; Williamon et al., 2013). These psychological and physiological stresses degrade the artistic quality of the performance (e.g., Yoshie et al., 2009b).

The phenomenon of performance anxiety, however, contradicts performers' self-reported positive experiences of live performance: “I always tell my orchestra it is not enough to be perfect on [studio] recordings. It must be like a live performance which will bring people up from their seats,” a comment by the German conductor Kurt Masur (Badal, 1996, p. 37). This is what Zajonc (1965) called “social facilitation,” a well-established theory that human (or animal) performances are facilitated by the presence of others when the performer is skilled in the target task and when the task is simple enough for the performer (Strauss, 2001). This suggests that the quality of music performance can also be enhanced while performed live, at least, when skilled performers play a familiar piece. The purpose of the present study was to test this hypothesis in piano performance.

**Figure 1:**
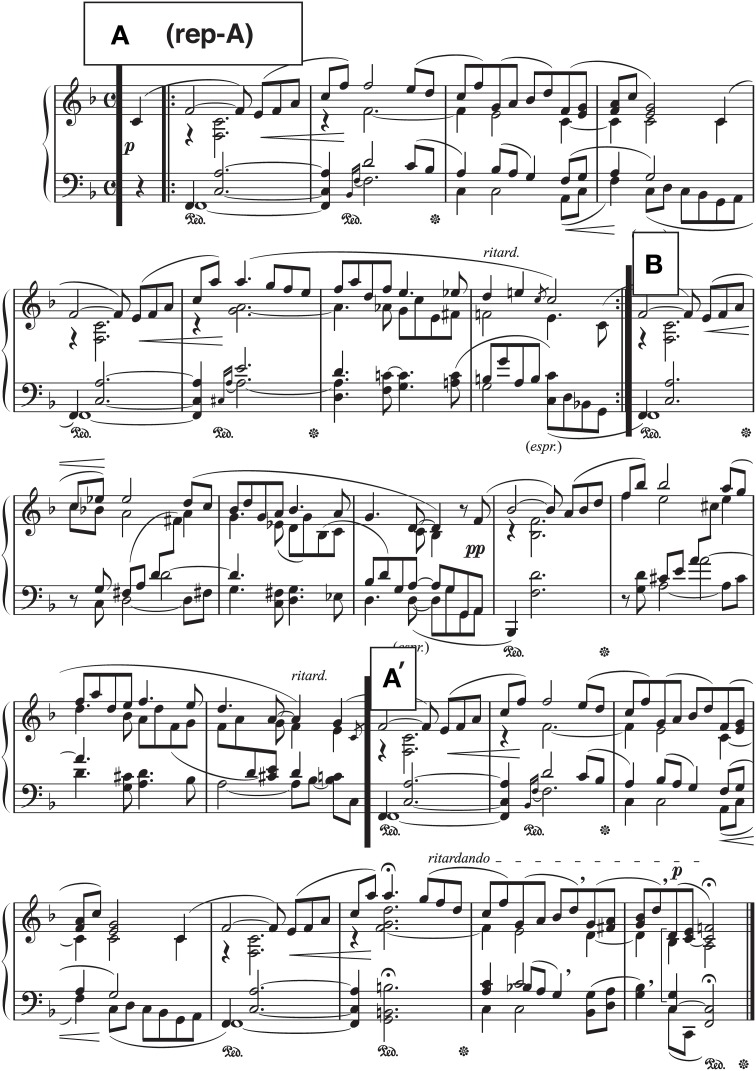
**The score of “*Träumerei*,” used by all the pianists**. Section IDs (boxed letters) are added for this Figure. This image was reproduced by the first author.

The present research consisted of three studies. In Study 1, we tested whether social facilitation would exist in piano performance by means of 153 listeners' evaluations between the audience-present and the audience-absent recordings made by 13 pianists. Studies 2 and 3 were conducted to identify pianists' acoustical expressions differing between the two recordings and to determine how acoustical differences would explain listeners' evaluations, respectively. To investigate a possible social facilitation in live recording, we chose a piece that is not technically demanding (Strauss, 2001) but requires rich expressions: *Träumerei*, the seventh of Robert Schumann's *Kinderszenen* (“Scenes from Childhood,” Op. 15). This piece consists of three 8-bar sections (A, B, A′) with the obligatory repetition of the first section (rep-A): It consists of a number of repetitions and variations of a 4-bar phrase including a modulation in B section (Monma, 1957; Figure 1). As performance practice for a piece from the Romantic period (Palmer and Halford, 1978), pianists employ the overall *tempo rubato* (i.e., a musical term for temporal liberty from the score) in addition to *ritardando* (or *ritard*.) at the end of each section even though *tempo rubato* is not indicated in the score (see Figure 1). We explored the mechanism of social facilitation in pianists' performances of this particular piece.

## Study 1

To investigate the existence of social facilitation in piano performance, we compared listeners' evaluations of performances recorded in front of an audience (“audience-present recording”) and those recorded alone (“audience-absent recording”). We assessed the following two aspects that can represent general impressions to music: *quality* (“how good the recording sounded”) and *emotionally moving experience* (“how much the audience was moved by the recording”).

A *quality* scale measures the overall quality of performance including both technical and expressive aspects. When this scale is used to assess multiple versions of the same piece, listeners' evaluations may reflect their preferences (Clarke, 1993). An *emotionally moving experience* scale measures the degree of emotional experiences that can be recognized physically such as goose pimples, lump in the throat, and shivers down the spine; and psychologically such as feeling sad, happy, elevated, vigorous, and/or dreamy (Yasuda et al., 2007). Music psychologists consider this experience different from the listener's perception of the quality of the performance (e.g., Gabrielsson, 2001-2002). We hypothesized that music recorded in front of an audience would sound better and emotionally move listeners more than the audience-absent recordings, in line with the theory of social facilitation (Zajonc, 1965; Bond and Titus, 1983; Strauss, 2001).

### Method

#### Participants

Participants were 153 graduate and undergraduate students (61 men, 92 women, 18–45 years old, *M* = 20.82, *SD* = 2.87), who had served as audience members in one of 13 pianists' live recording sessions (see Section Stimuli below) 10 weeks before the current experiment. All had heard Schumann's *Träumerei* prior to the live recording session. Participants were not majored in music but had at least 9 years of classroom music instruction including music appreciation as a compulsory school education. The years of private musical training and/or extracurricular activities (e.g., piano, clarinet, guitar, violin, choir, brass band, orchestra, improvisation, composition) ranged from 0 to 19 years (*M* = 10.37, *SD* = 6.14). Participants were categorized into three groups based on the years of musical training: least experienced (i.e., 0–5 years of musical training, *n* = 37), moderately experienced (i.e., 6–15 years, *n* = 77), and most experienced (i.e., 16–19 years, *n* = 39). Participants received 500 JPY (approximately U.S. $5.00) as an incentive upon the completion of this experiment. The informed consent was obtained from each participant prior to the experiment, following ethical standards suggested by American Psychological Association (2009).

#### Stimuli

Thirteen pianists (4 men, 9 women, 24–40 years old, *M* = 30.46, *SD* = 4.41) with a music degree recorded their performances both with and without the audience. They were a concert pianist (*n* = 1), lecturers at a university or a vocational school (*n* = 4), piano teachers at private music institutions (*n* = 7), and a music therapist at a hospital (*n* = 1). They started to play the piano between ages 4 and 6. We selected six Western-classical pieces as the materials, including *Träumerei* (Figure 1). All the pianists reported that they had performed *Träumerei* many times since childhood. We asked pianists to practice these pieces at least for 1 month before the recordings.

Recordings took place in a small auditorium (with the maximum capacity of 114), equipped with a grand piano (GP-193, Boston). The sound pressure level of background noise was 33.4 dBA, measured by a sound-level meter (DT-8852, MK Scientific). The piano was tuned professionally within 1 week before each pianist's recording.

On the day of recording, pianists rehearsed each piece as many times as they wished before the performance. Subsequently, each pianist performed six pieces in a random order specified by the experimenter (H.S.) in front of 11–23 participants (audience-present context). Each pianist performed the same pieces in the same order either before (*n* = 6) or after (*n* = 7) the live performance without any audience (audience-absent context). Performers could re-record the performance as many times as they wished in the audience-absent context. The performances were stereo-recorded onto a multi-track recorder (R24, Zoom) using a microphone (NT4, Rode). The performance portion of the recording lasted approximately 45 (audience-present) and 30 min (audience-absent). The first author confirmed by listening to all the recordings that no noise from the audience was audio-recorded.

#### Procedure

Ten weeks after they attended the live recording of the pianist's performances, participants returned to the same auditorium, and listened to both the audience-present and the audience-absent recordings of six pieces played by the same pianist, individually or in a group of 2–10. We considered the insertion of 10 weeks after the participant's initial exposure to the live performance to be enough to eliminate a possibility of a mere-exposure effect (i.e., an effect that multiple exposures increase one's preference), which can disappear in 1 month (Peretz et al., 1998). The order of recording contexts was counterbalanced. The order of stimuli in the first block was randomized for each pianist and the same stimulus order was used in the second block. In other words, each participant listened to both the audience-present and the audience-absent recordings in the same order. Stimuli were presented by a stereo speaker (WS-AT30, Panasonic) through an amplifier (RX-V603, Victor) and a computer (MC505J/A, Apple). Participants rated each piece on a 9-point Likert scale (1 as “not at all” to 9 as “extremely”) for each of the Japanese equivalents of the two items: “good” (*yokatta*) and “emotionally moving” (*kando-shita*). After evaluating all the pieces, participants provided their background information (i.e., age, sex, years and types of musical training).

### Results and discussion

For the purpose of the present study, we focused on the participants' evaluations of *Träumerei*. Because a preliminary analysis (one-way between-subject analysis of variance) for neither the quality nor the emotionally moving experience yielded any significant main effect of the performer [*Fs*_(12, 140)_ = 1.18–1.51, *ps* = 0.13–0.31, η^2^_p_s = 0.09–0.11], the pooled data were used in the following analyses.

Table 1 shows the mean scores of the quality and the emotionally moving scales based on training. We conducted a 3 (i.e., training as between-subject) × 2 (i.e., recording condition as within-subject) mixed-design analysis of variance for each scale. For the quality (Table 1A), a main effect of the recording condition was significant, but that of the training and a two-way interaction were not significant, *F*_(1, 150)_ = 6.57, *p* = 0.01, η^2^_p_ = 0.04 (recording condition), *F*_(2, 150)_ = 1.44, *p* = 0.24, η^2^_p_ = 0.02 (training), *F*_(2, 150)_ = 1.79, *p* = 0.17, η^2^_p_ = 0.02 (two-way interaction). Listeners evaluated the audience-present performance (*M* = 6.77, *SD* = 0.12) better than the audience-absence performance (*M* = 6.47, *SD* = 0.13).

**Table 1:** **The mean scores of quality (A) and emotionally moving experience (B) by the least, the moderately, and the most experienced listeners**.

**Training**	**Audience-present**		**Audience-absent**	
	***M* (*SD*)**	**95% CI**	***M* (*SD*)**	**95% CI**
**(A) QUALITY**				
Least	6.86 (1.40)	[6.40, 7.33]	6.49 (1.73)	[5.91, 7.06]
Moderately	6.82 (1.37)	[6.51, 7.13]	6.79 (1.39)	[6.48, 7.11]
Most	6.62 (1.48)	[6.14, 7.10]	6.13 (1.58)	[5.62, 6.64]
**(B) EMOTIONALLY MOVING EXPERIENCE**				
Least	5.89 (1.82)	[5.28, 6.50]	5.70 (1.88)	[5.07, 6.33]
Moderately	6.21 (1.62)	[5.84, 6.58]	6.21 (1.61)	[5.84, 6.57]
Most	6.13 (1.70)	[5.58, 6.68]	5.46 (1.76)	[4.89, 6.03]

*CI, Confidence interval*.

For the emotionally moving experience (Table 1B), a main effect of the recording condition was significant and a two-way interaction was approaching significance, *F*_(1, 150)_ = 4.75, *p* = 0.03, η^2^_p_ = 0.03 (recording condition), *F*_(2, 150)_ = 2.46, *p* = 0.09, η^2^_p_ = 0.03 (two-way interaction). A main effect of training was not significant, *F*_(2, 150)_ = 1.40, *p* = 0.25, η^2^_p_ = 0.02. For the two-way interaction, *post-hoc* paired *t*-tests with Bonferroni's correction (overall α = 0.10, subset α = 0.10/3 = 0.033) revealed that the most experienced listeners were moved more by the audience-present than the audience-absent performance, *t*_(38)_ = 3.41, *p* = 0.002, *d* = 1.11. For the least and the moderately experienced listeners, no significant differences were found between the two performances, *t*_(36)_ = 0.50, *p* = 0.62, *d* = 0.17 (the least experienced), *t*_(76)_ = 0.00, *p* = 1.00, *d* = 0.00 (the moderately experienced).

The above results confirmed that listeners evaluated the audience-present better than the audience-absent recording regardless of their level of musical training, as found in their quality ratings. This is the first empirical evidence that social facilitation (Zajonc, 1965) can exist in the domain of music performance, at least, when the expert performer records a familiar, simple piece (such as *Träumerei*). As Yoshie et al. (2009a) reported, the artistic quality of the performance can decrease in front of judges in a competition, in which pianists perform technically challenging pieces. Thus, the general theory of audience effect (Bond and Titus, 1983; Strauss, 2001) seems applicable to music performance: The difficulty of the task determines whether the presence of the audience leads to social facilitation or inhibition.

As for the emotionally moving experience, social facilitation was evident only in most musically experienced listeners, perhaps because their emotional sensitivity to music and/or some personality aspects (e.g., absorption to music, Sandstrom and Russo, 2013) might influence their emotional experience. It is possible, then, that the mechanism of social facilitation in the emotionally moving experiences is different from that in the perception of quality of the performance. We explore this issue by incorporating the acoustical measurements of the performances in Studies 2 and 3.

## Study 2

One of the goals of music performance is communication of the structure of the piece, reflecting the composer's idea (Clarke, 1985; Palmer, 1996; Friberg and Battel, 2002). Performers can communicate with the audiences by highlighting their expressions along with the musical structure. For example, Repp (1992, 1995) has shown that the tempo of *Träumerei* varies in accordance with the hierarchical structure of the piece: Pianists frequently lengthen accented tones within melodic gestures, and this lengthening is more exaggerated at the end of each section in accordance with the composer's *ritardando* marking (i.e., A, rep-A, B, A′; see Figure 1). The degree of lengthening is the greatest at the end of the piece (i.e., the end of the “highest” level in the structural hierarchy of the piece).

Despite the valuable contributions made by Repp (1992, 1995), his original analyses treated each note as a different variable, ignoring that notes being closer in the score are more statistically related than those being farther away (Almansa and Delicado, 2009). To solve this problem, Almansa and Delicado (2009) applied their proposed time-series analyses to the expressive timing data of Repp's (1992), measuring the duration for each eighth note of the piece while treating each performance as a continuous function of one variable. Using functional principal components analysis (Ramsay et al., 2009; see Section Time-series analyses of pianists' durational expressions for the audience-present and the audience-absent performances), they found that the timing structure of *Träumerei* consisted of five components, two of which explained over 80% of the timing variability of this piece. The first component (60.30% of total variability) represented the global tempo, meaning that the main durational expression of the piece was determined by how much faster or slower pianists performed. The second component (20.02% of total variability) represented the temporal contrast between the final portion and the rest of the piece, representing the pianist's tendency to play the final portion more slowly than any other portion (Repp, 1992). The remaining three components explained more local features of the timing variation (5.00%, 4.53%, and 2.18% of total variability for the third, the fourth, and the fifth components, respectively). For example, their third component showed the typical expression at the transitional point from sections B to A′ or the duration of the first *fermata* in section A′ (see Figure 1). More specifically, when sections A and B were performed at a faster tempo, pianists tend to slow down more at the transition from sections B to A′ as well as to lengthen the first *fermata* in section A′. In other words, Almansa and Delicado's analyses indicate that the timing structure of *Träumerei* is determined in global and local ways, such as the global tempo (the first component), the temporal contrasts between the final and the remaining sections (the second component, i.e., the “global” variability reflecting the structure of the piece), and the timing deviation at the specific instruction by the composer (the remaining components, i.e., the “local” variability such as *fermata*). However, the first two components contributing over 80% of the total variability appear to suggest that they are the primary source of the pianist's durational expressions. In the present study, we examined whether the same two components could be replicated in addition to how pianists differentiated each component between the recording contexts.

We also conducted time-series analyses on pianists' dynamic expressions. Previous studies show the correlation between the temporal and the dynamic expressions such as “the faster, the louder” and “the slower, the softer” (Todd, 1992; Repp, 1996). If this were true, we would extract the same components for both the durational and the dynamic expressions, and pianists' differentiations between the recording contexts would also be consistent.

### Method

Participants (i.e., pianists), apparatus, and procedure of the recordings were reported in Study 1 (see Section Stimuli). We would describe measurement and computation of parameters below.

For the present study, we measured duration and dynamics in the following. After transforming the digital recording of each performance to the voltage waveform by the sound visualization software (Wavosaur, Wavosaur Team), the first author manually identified the onset of each beat and computed the duration of each quarter note (“beat duration”). As for dynamics, the A-weighted sound level (dBA) per 3.33 ms was captured by a 1/3 octave band analysis (DSSF 3.5.1, Yoshimasa Denshi), which corresponds with human perception of loudness of complex tones (Stevens, 1955). In order for the sound level data to be synchronized with the beat, we identified the peak sound levels at the loudest and the softest keystrokes within a beat, and calculated the difference between them (“dynamic range” per beat). Note that the sound level at the softest keystroke is not influenced by the decay of piano tone unlike the minimal sound level within a beat. Both the minimal and the maximal keystrokes can be considered to be expressive manipulations controlled by the pianists. For this reason, we believe that the within-beat difference of these values (i.e., dynamic range) is an appropriate index in comparing the degree of expressivity among multiple versions of the same piece (Shoda and Adachi, 2010). The mean of the beat duration and that of the dynamic range represent the overall tempo and dynamic range, respectively. We analyzed the variations of these parameters as well. As a parameter of “overall” variations within a performance, we calculated the “coefficient of variation” (the standard deviation normalized by the mean value). As a parameter of the “cross-sectional” variations per performance, we first calculated a range of each parameter (i.e., a difference between the maximal and the minimal values) within each section of the piece, and then obtained the standard deviation of those ranges.

Our measurement neither used MIDI format nor treated an eighth note as a unit of analysis, as did previous studies (Repp, 1992; Almansa and Delicado, 2009). This was because a MIDI console was unavailable on our Boston piano, and we decided to go in line with Repp (1990), who identified the onset of each beat manually, as mentioned above. Moreover, we believed that pianists would manipulate their expressions based not on the shortest note but on the beat of a piece (i.e., a quarter note in 4/4 time) as shown in Shoda and Adachi (2012). To confirm the reliability of the identification, two volunteers independently confirmed the accuracy of the first author's measurements of the beat duration for all the recordings.

### Results and discussion

#### Basic statistics

We computed mean, coefficient of variation, and cross-sectional variation of the duration and the dynamic range for each performance (Table 2). To examine effects of the recording context on these parameters, we conducted permutation paired *t*-tests. A permutation test is an alternative way to examine differences in population parameters in a non-parametric fashion, so that we do not need to make any assumption about the sampling distribution (e.g., normal distribution) of the test statistic (Good, 2005). Moreover, permutation tests allow us to use raw data unlike conventional non-parametric methods (e.g., Wilcoxon signed rank test) that require transformation of original data to another form such as rank (Good, 2005). We computed a *t*-statistic of the obtained observations (*t*_obt_), and then, the sampling distribution of the *t*-statistic was generated by means of 10,000 iterations of permutated data. A *p*-value was obtained by computing a proportion of the iterations that was equal to or greater than the actual grouping of the data. The tests showed no significant differences between the conditions with small effect sizes (*ps* = 0.39–0.79, *ds* = 0.04–0.19), indicating that pianists' expressions of the duration and the dynamics were not differentiated between the recording conditions. However, the calculation of the parameters from the beginning to the end of the piece might lose information such as pianists' particular expressions at structurally important locations (e.g., Repp, 1990, 1992, 1995, 1996). In addition, such time-series expressions might differ as a function of pianist. To solve these problems, we conducted a functional principal components analysis (Ramsay et al., 2009) to identify the time-series features of *Träumerei* performances across individual pianists.

**Table 2:** **The mean (*M*), the coefficients of variation (*CV*), and the cross-sectional variations (*CSV*) of the duration and the dynamic range for the audience-present and the audience-absent conditions across 13 pianists**.

	**Audience-Present *M* (*SD*)**	**Audience-Absent *M* (*SD*)**	*t*_obt_^*^	p^**^	**Cohen's *d***
**DURATION (s)**					
*M*	52.30 (3.98)	53.14 (4.44)	0.68	0.67	0.13
*CV*	0.25 (0.03)	0.25 (0.03)	0.64	0.53	0.08
*CSV*	4.47 (1.76)	4.78 (1.58)	0.93	0.39	0.19
**DYNAMIC RANGE (dBA)**					
*M*	16.40 (0.91)	16.36 (1.06)	0.27	0.79	0.04
*CV*	0.29 (0.03)	0.29 (0.03)	0.83	0.43	0.11
*CSV*	3.18 (1.41)	3.05 (1.84)	0.31	0.75	0.08

* *df = 12*.

** *The p values were computed by permutation paired t-tests*.

#### Time-series analyses of pianists' durational expressions for the audience-present and the audience-absent performances

**Figure 2:**
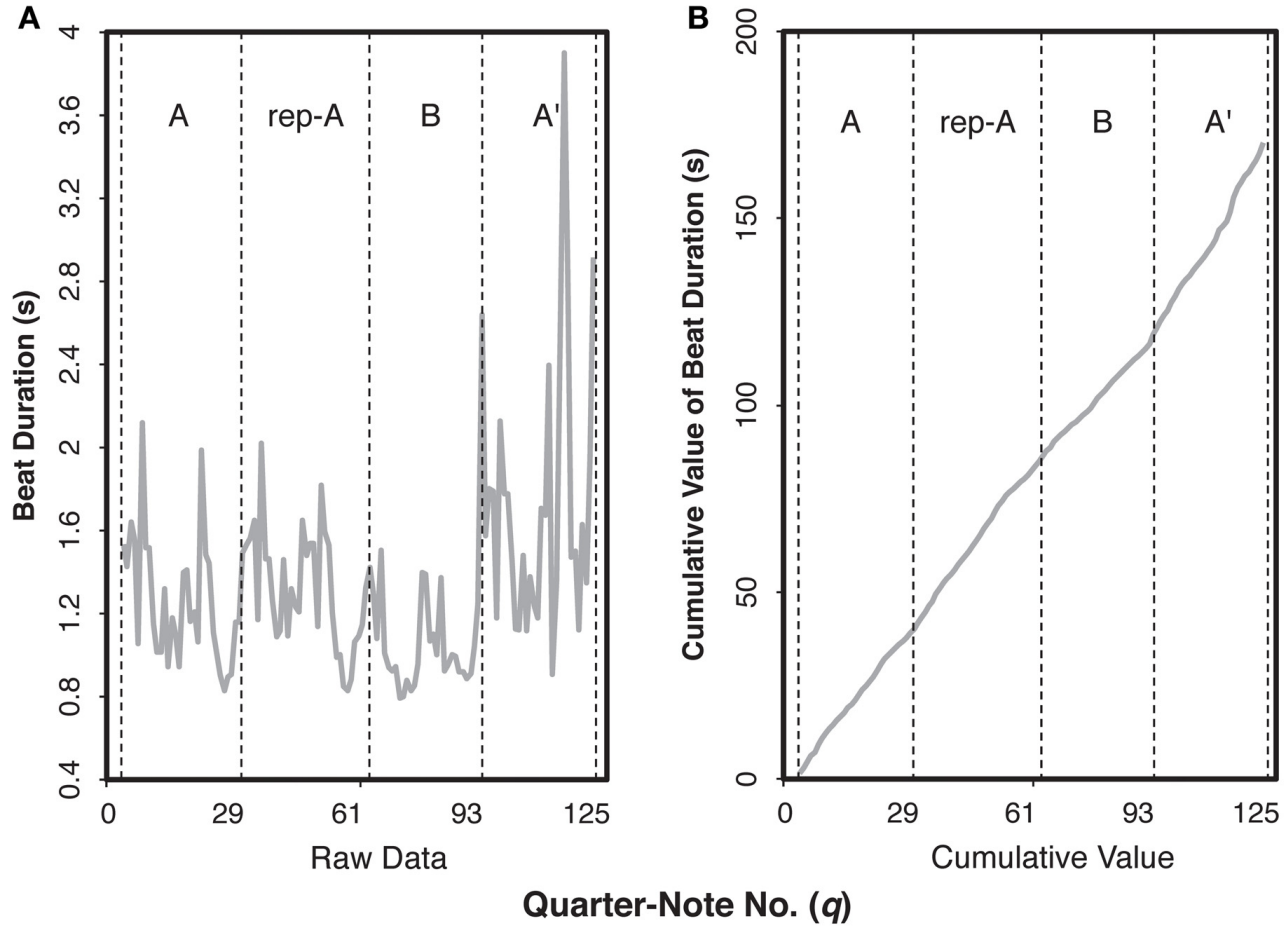
**Examples of the raw data (A) and the cumulative value (B) for the beat duration of the audience-present performance by Pianist 11**. The letters within each graph indicate section IDs. The x-axis indicates the number of quarter note.

Time-series analyses were conducted with FDA and STATS packages in R. The following analyses were based on the method by Almansa and Delicado (2009). First, we calculated the smoothed beat duration by computing the cumulative value from the beat duration data. Figure 2 shows an example of the raw data and the cumulative values by Pianist 11, who showed the clearest difference between the audience-present and the audience-absent conditions. The cumulative value can be expressed continuously: For any quarter-note number *q* ∈ [1, 127] we can estimate the elapsed time, *t*_(q)_, by smoothing of the data (e.g., linear interpolation). Note that the inverse of the elapsed time function is equivalent with the position function in physics, *q*_(t)_, indicating that we can estimate the position of the score (*q*) by the elapsed time (*t*). The position function might be important in physics, for we can estimate the velocity and the acceleration by calculating derivatives of the position function. However, it is likely that pianists manipulate tempo and dynamics based on notes on the score rather than clock time (Almansa and Delicado, 2009). Thus, variations as a function of score position are musically more important than those of elapsed time.

**Figure 3:**
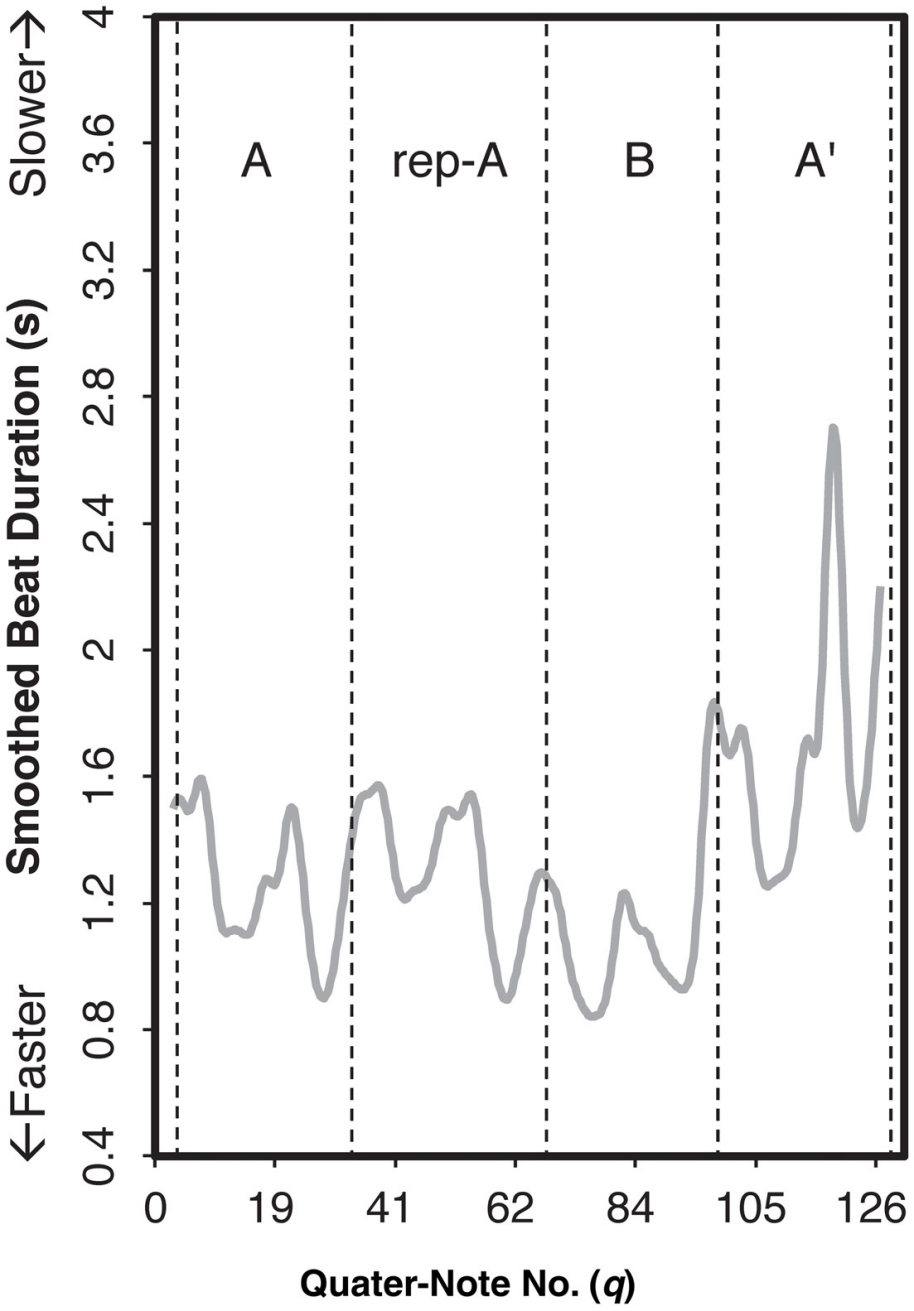
**Examples of the smoothed beat duration curves for the audience-present performance by Pianist 11**. The letters within the graph indicate section IDs. The x-axis indicates the number of quarter note.

In the present study, the smoothing of the cumulative value was conducted by a non-parametric regression method. According to Almansa and Delicado (2009), performance parameters (e.g., timing, dynamics) of a musical piece does not fit well by means of a closed parametric function that can be expressed analytically only with the finite number of elementary functions because of variability within a single performance. We applied local polynomial regression with degree two (see Fan and Gijbels, 1996) for the cumulative values of each performance (Figure 2B) and calculated the first derivative as the smoothed beat duration (Figure 3 as an example). A higher value in the curve at a quarter-note position *q* means that the note at *q* took more time to be played and consequently its real velocity was lower.

As shown in Figure 3, the beat duration was slower in section A′ (the end of the piece) than in the other sections, indicating that the pianist modulated the beat duration based on the overall structure of the piece. This can be considered as “global” variation. Moreover, the patterns of *accelerando* (i.e., playing faster and faster) and *ritardando* (i.e., playing slower and slower) were observed periodically within a section, which can be considered as “local” variation. Thus, in line with previous studies (e.g., Repp, 1992, 1995; Almansa and Delicado, 2009), the durational expression of *Träumerei* appeared to be characterized by both global and local variations.

**Figure 4:**
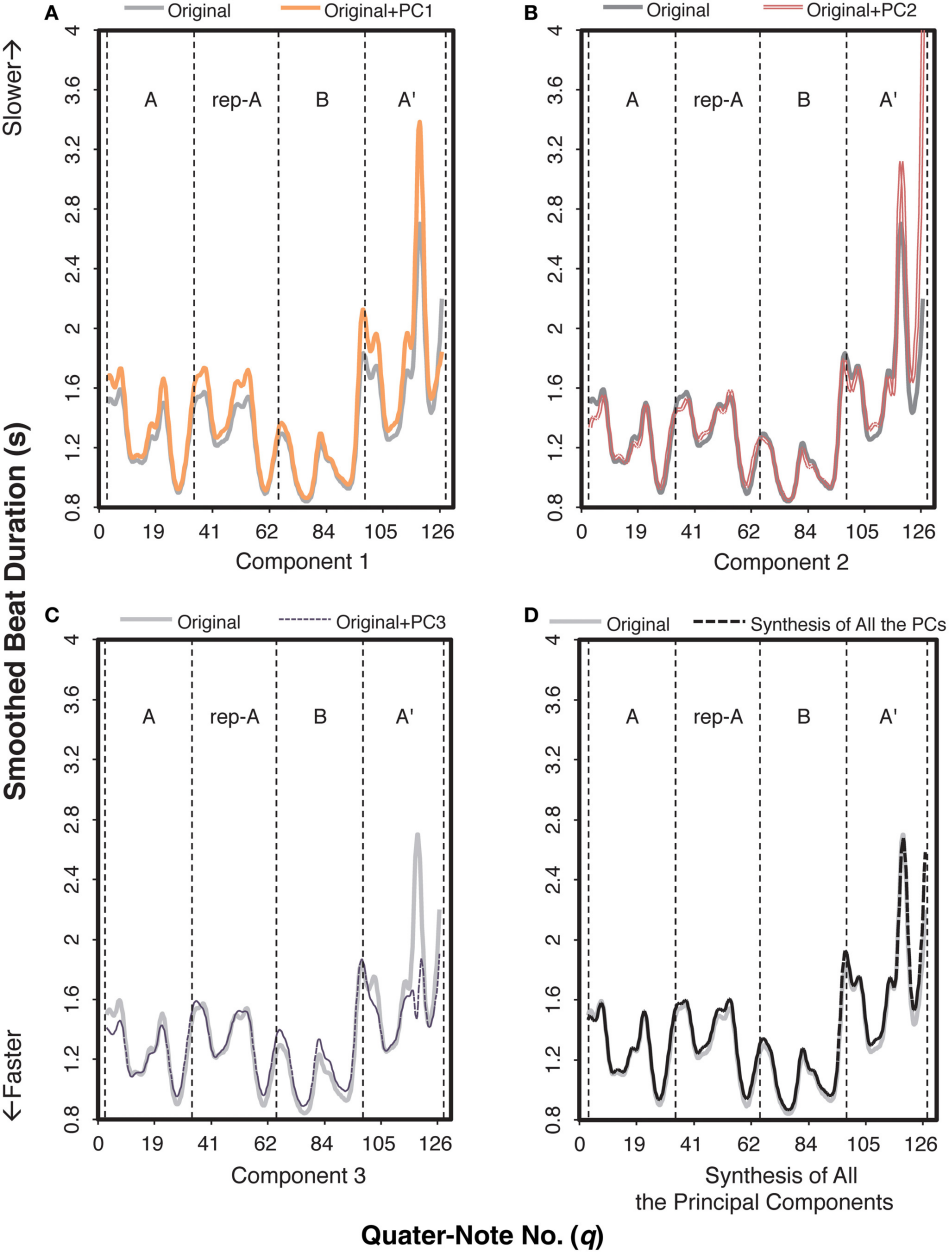
**An example of how each component functioned in the smoothed beat duration of the audience-present performance, based on Pianist 11's data**. The role of each component can be extracted by adding the value of the target component to the original curve. The letters within each graph indicate section IDs. The x-axis indicates the number of quarter note.

To identify durational variations differing between the two contexts, we first applied a functional principal components analysis (Ramsay et al., 2009), revealing multiple components in the durational expressions of *Träumerei* executed by the pianists. Results showed three components (PCs; Supplementary Figure S1) in the smoothed beat duration that were responsible for these durational variations. A total of 76.59% of variance was accounted for by these components: 42.86% (PC 1), 17.77% (PC 2), and 15.96% (PC 3). Figure 4 shows an example of what each component contributed to the smoothed beat duration by adding each principal component to the original curve. Because the synthesis of the three components was fairly identical to the original curve (Figure 4D), we can conclude that the beat duration was explained to a large extent by these components.

**Figure 5:**
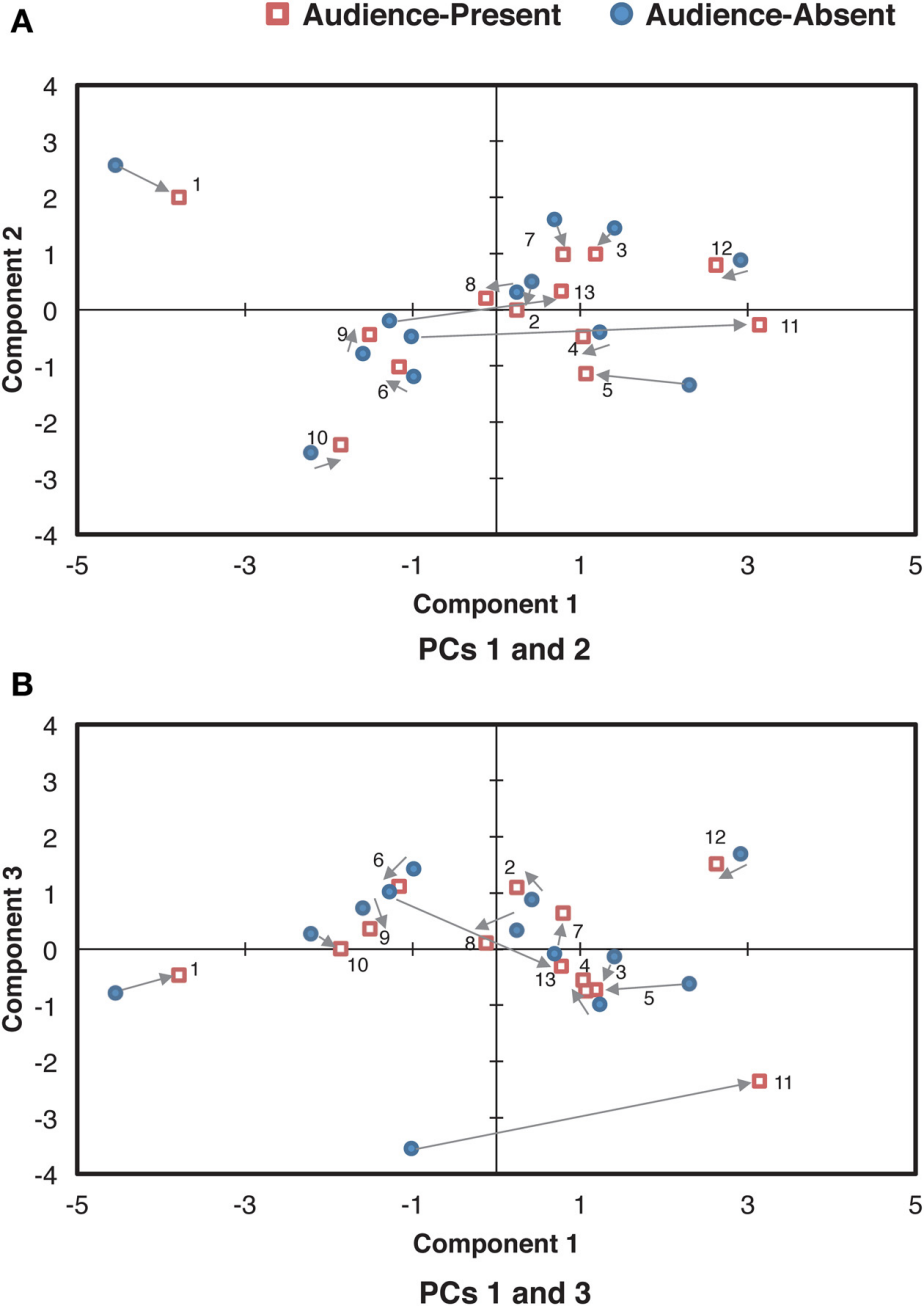
**Relationships of the component scores between PCs 1 (the overall mean) and 2 (the cross-sectional contrast between the final and the remaining sections) (A) and between PCs 1 and 3 (the control of overall variability) (B) for the smoothed beat duration**. The arrows indicate the directions changed from the audience-absent (•) to the audience-present (□) conditions. The numerals in the plots are pianist IDs. (PC: principal component).

More specifically, PC 1 generally increased the values of the original curve (Figure 4A); the role of PC 1 can be regarded as the amplification of the overall beat duration. Based on the component score of PC 1 (“PC 1 score”), we can categorize the performances as a function of the overall tempo. For example, in Figure 5, PC 1 score of the audience-present performance by Pianist 11 was the highest, meaning that he performed this piece in the slowest tempo (i.e., 48.88 bpm based on the mean beat duration) among the pianists. In contrast, PC 1 score of the audience-absent performance by Pianist 1 was the lowest, meaning that she performed it in the fastest tempo (i.e., 63.05 bpm based on the mean beat duration). The high correlation (*r* = 0.97, *p* < 0.001) between the PC 1 score and the mean beat duration (mean value of the smoothed beat duration calculated from the beginning to the end of the piece for each pianist) also verifies that PC 1 indicates the overall beat duration, or the overall tempo.

When PC 2 was added (Figure 4B), the values in sections A, rep-A, and B were relatively consistent to the original curve, but those in section A′ were enhanced, especially at the very end. This indicates that this component functioned as the temporal contrasts between the final and the remaining sections, reflecting global structure of the piece.

The most prominent role of PC 3 (Figure 4C) was the extreme reduction of the value at *fermata* before the final *ritardando* (see Figure 1, measure 22, *q* = 116), resulting in a small range of the variation in section A′. While the pianists originally performed section B in a relatively faster tempo, PC 3 made this section slower. It appears that PC 3 made the range of the curve more consistent across sections, implying that PC 3 was controlling the temporal variability throughout the piece.

Next, we analyzed how these components contributed to differences in the individual pianists' durational expressions between the audience-present and the audience-absent performances. The component score in each condition for each pianist was scatter-plotted in Figure 5. The larger value indicates the greater contribution of each component to the corresponding performance. Zero value represents the mean of each component, indicating that the pianist's expression in the target component is on average. As for the difference between the conditions, most plots in the audience-present condition were closer to “zero” as compared with those in the audience-absent condition. To confirm this, we conducted a permutation paired *t*-test and compared the absolute values (representing the distance from “zero”) between the two conditions for each principal component. The absolute value in the audience-present condition (*M* = 0.85, *SD* = 0.68) was significantly smaller than that in the audience-absent condition (*M* = 1.10, *SD* = 0.76) for PC2, *t*_obt_(12) = 3.74, *p* = 0.002, *d* = 0.33, but the tests for PCs 1 and 3 did not yield significant differences, *t*_obt_(12) = 0.56, *p* = 0.63, *d* = 0.11 (PC 1), *t*_obt_(12) = 1.44, *p* = 0.18, *d* = 0.26 (PC 3). These results indicate that the pianists expressed the durational contrasts between the final and the remaining sections (PC 2) in a more “averaged” (or less unique) manner in the audience-present conditions.

The functional principal components analysis has shown that the durational expression of *Träumerei* consists of three components representing the overall tempo (PC 1), the temporal contrast between the final and the remaining sections (PC 2), and the control of the temporal variability (PC 3). The first and the second components in the present study were concordant with those in Almansa and Delicado (2009), who analyzed *Träumerei* recorded in a laboratory setting (i.e., in front of one experimenter). This reconfirms that the overall tempo and the temporal emphasis of the ending section are two general principles in the temporal expression of this piece, as shown in previous studies (e.g., Clarke, 2001; Repp, 1992). None of the remaining components in Almansa and Delicado (2009) corresponded to our third component (i.e., controlling the temporal variability within a piece), possibly because they used an eighth note rather than a quarter note as the unit of analysis.

Additionally, the pianists were likely to express a more “averaged” temporal contrast between the final and the remaining sections in the live recording. This appears to indicate that pianists in the live recording context control themselves such that they do not overdo the structural expressions in communicating their own artistry to the audience, in line with the literature (e.g., Shoda and Adachi, 2010, 2012).

#### Time-series analyses of pianists' dynamic expressions for the audience-present and the audience-absent performances

**Figure 6:**
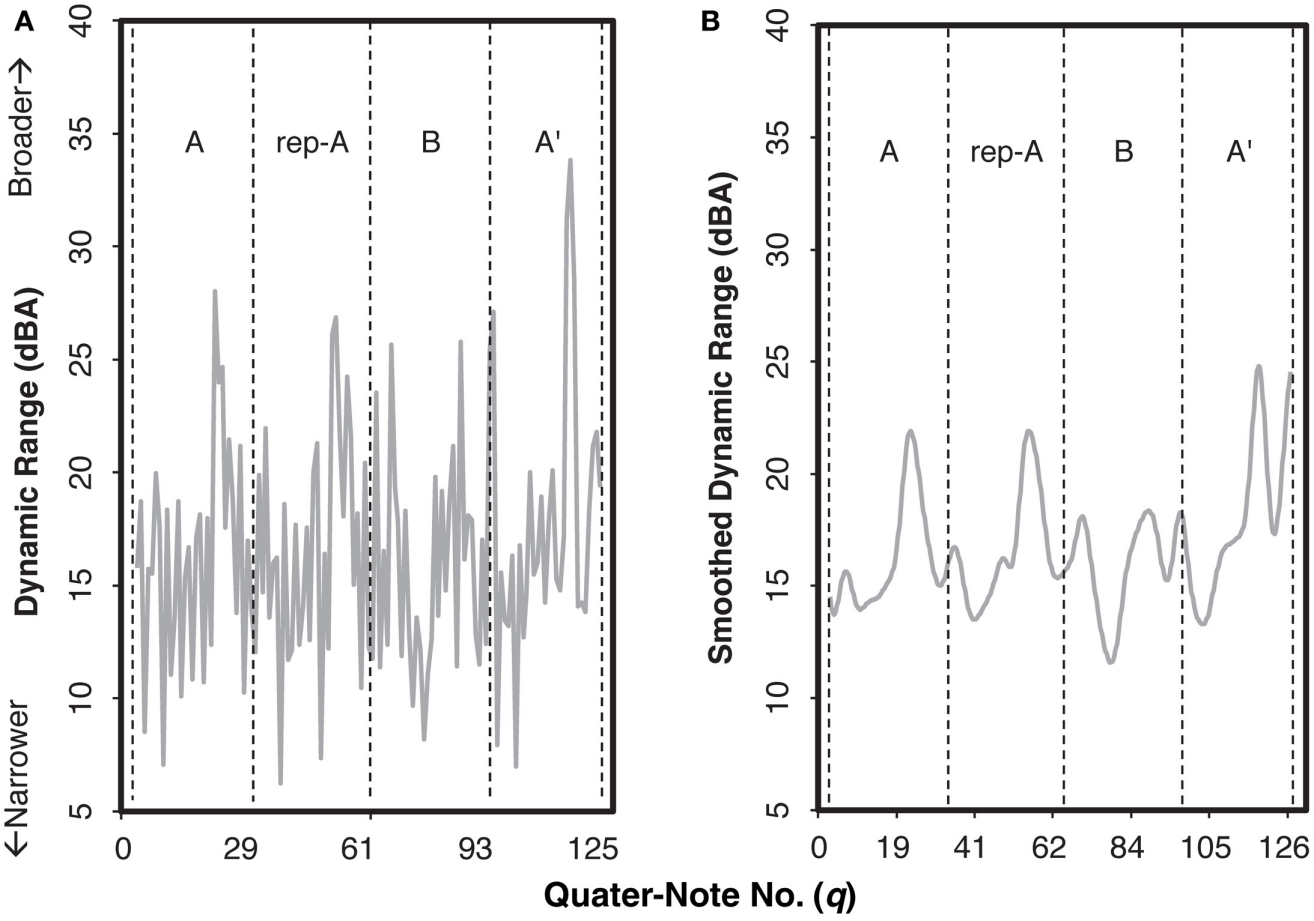
**Examples of the original dynamic range (A) and the smoothed curve as the smoothed dynamic range (B) for the audience-present performance by Pianist 11**. The letters within each graph indicate sections IDs. The x-axis indicates the number of quarter note.

For the dynamic range (Figure 6A), we also applied the same time-series analyses. An example of the smoothed curve (Figure 6B) indicates the degree of change in dynamic range as a function of beat; we shall call this curve as a “smoothed dynamic range” curve. Similar to the smoothed beat duration, the smoothed dynamic range appeared to reflect the structure of the piece. In Figure 6B, the general patterns in sections A, rep-A, and A′ were similar, in that the highest peak exists around two third of each section. Nonetheless, that peak was the highest in section A′, indicating that pianists differentiated the ending section of the piece from the others sharing the same theme. The pattern of section B, having two low peaks and a deeper trough, was different from any other section, highlighting its function as the contrastive (or the development) section of the piece.

**Figure 7:**
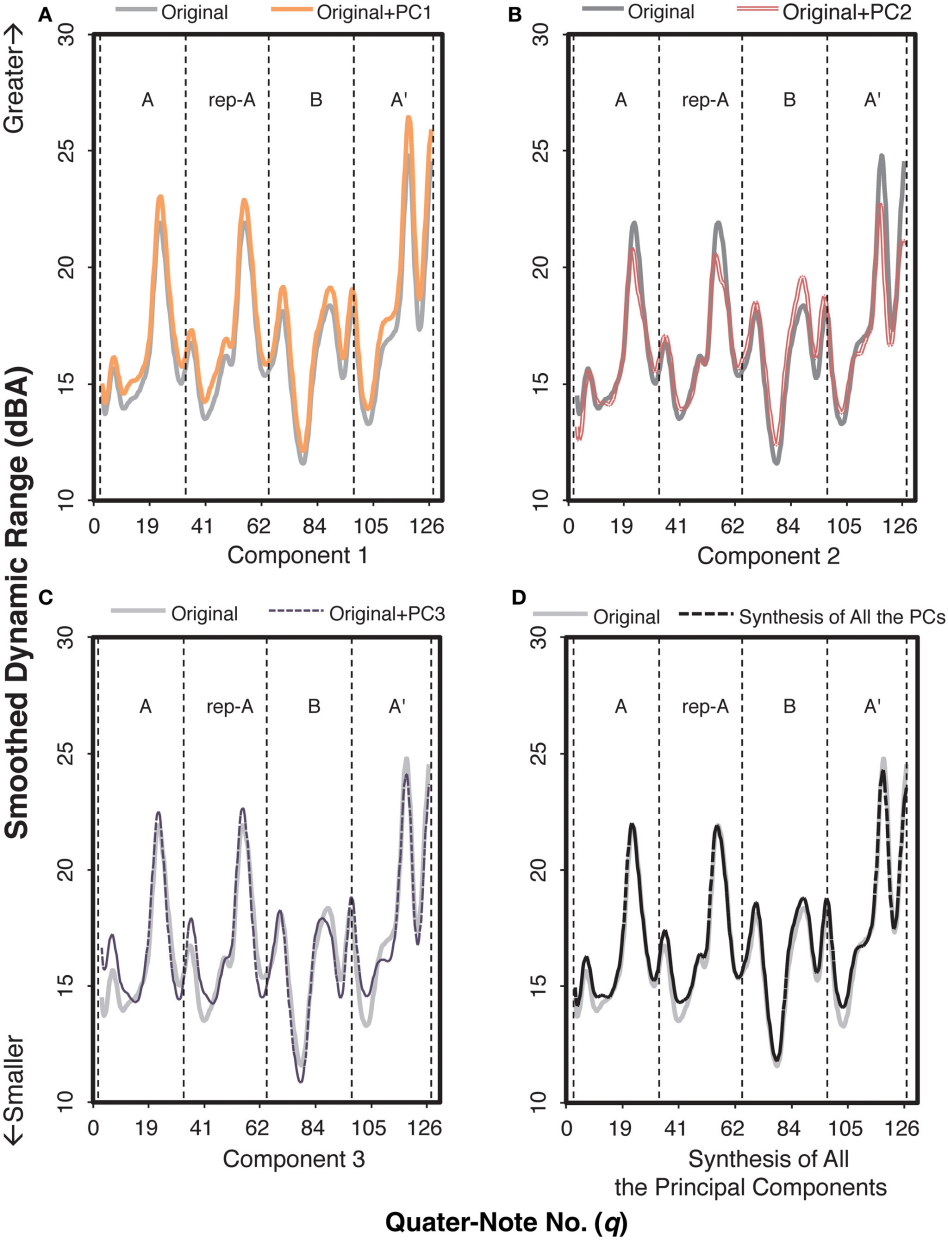
**An example of how each component functioned in the smoothed dynamic range curve of the audience-present performance, based on Pianist 11's data**. The role of each component can be extracted by adding the value of the target component to the original curve. The letters within each graph indicate section IDs. The x-axis indicates the number of quarter note.

We conducted a functional principal components analysis to the smoothed dynamic range curve for each recording, extracting three principal components (Supplementary Figure S2). A total of 70.78% of the variance was accounted for by these components: 41.96% (PC 1), 17.05% (PC 2), and 11.76% (PC 3). Figure 7 shows an example of how each of the components functioned in the smoothed dynamic range curve by adding each component to the original curve. As can be seen in Figure 7D, the synthesis of all the components was fairly identical to the original curve, validating that the smoothed dynamic range curve was explained to a large extent by these three components.

By adding PC 1 to the original smoothed dynamic range curve, the value increased throughout the piece (Figure 7A), meaning that PC 1 enhanced the degree of the smoothed dynamic range curve. This was verified by the high correlation between the component score of PC 1 (“PC 1 score”) and the mean smoothed dynamic range (the mean value calculated for the smoothed dynamic range curve for each pianist), *r* = 0.96, *p <* 0.001.

PC 2 (Figure 7B) appeared to reflect the variation in the smoothed dynamic range curve. PC 2 represents the overall reduction of the range of the curve throughout the piece, that is, relatively higher peaks in sections A, rep-A, and A′ were suppressed while relatively lower peaks of section B were amplified. This was confirmed by the negative correlation between PC 2 score and the variation of smoothed dynamic range (the variation of the curve calculated from the beginning to the end of the piece), *r* = −0.91, *p <* 0.001.

The tendency of PC 3 (Figure 7C) appeared to differ as a function of the section (i.e., A/rep-A, B, A′). When PC 3 was added, the values generally increased except at the closing point of sections A and rep-A, whereas the values decreased at the troughs and the second peak while increasing at the closing point in section B. This indicates that in sections A, rep-A, and B′, PC 3 represents the pianist stretching out the smoothed dynamic range, particularly in comparing with the closing point. On the other hand, in section A′, PC 3 represents the smaller range of the curve, i.e., the first trough being higher and the peak at *fermata* (*q* = 116) being lower. The different tendencies between the final (A′) and the remaining sections imply that PC 3 reflected the pianists' expressions for the global structure of the piece.

**Figure 8:**
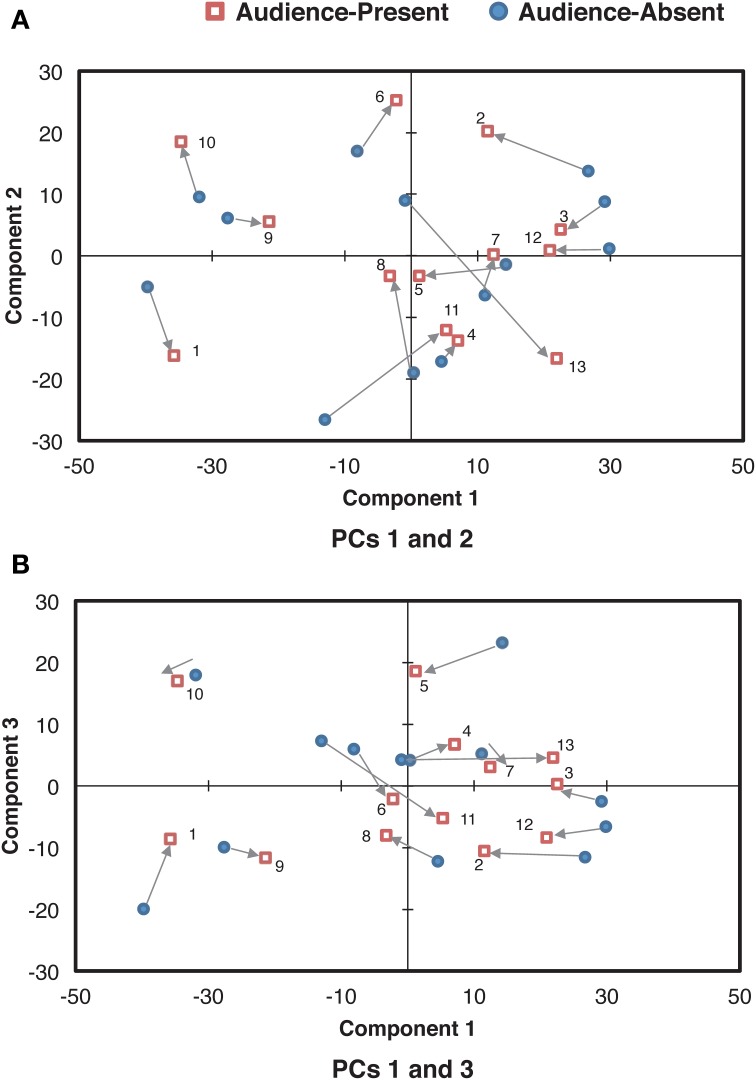
**Relationships of the component scores between PCs 1 (overall mean) and 2 (the control of overall variability) (A) and between PCs 1 and 3 (the cross-sectional contrast between the final and the remaining sections) (B) for the smoothed dynamic range curve**. The arrows indicate the directions changed from the audience-absent (•) to the audience-present (□) conditions. The numerals in the plots are pianist IDs. (PC: principal component).

Next, we analyzed how the individual pianists differentiated these components between the audience-present and the audience-absent performances. The component score in each condition for each pianist was scatter-plotted in Figure 8. As for the difference between the conditions, most plots in the audience-present condition were closer to “zero” (i.e., “average”) as compared with those in the audience-absent condition. To confirm this, we conducted a permutation paired *t*-test, comparing the absolute values between the two conditions for each principal component. The absolute value in the audience-present condition (*M* = 8.06, *SD* = 5.42) was significantly smaller than that in the audience-absent condition (*M* = 10.06, *SD* = 6.61) for PC 3, *t*_obt_(12) = 1.98, *p* = 0.05, *d* = 0.33, but the tests for PCs 1 and 2 did not yield significant differences, *t*_obt_(12) = 1.10, *p* = 0.29, *d* = 0.23 (PC 1), *t*_obt_(12) = 0.02, *p* = 0.98, *d* = 0.01 (PC 2). These results indicate that pianists differentiated between the final and the remaining sections by manipulating the variability in the smoothed dynamic range (PC 3) in a more “averaged” (or less unique) manner in the audience-present conditions.

The dynamic expression consisted of the overall degree of smoothed dynamic range (PC 1), the overall control of dynamic variability (PC 2), and the cross-sectional contrast between the final and the remaining sections (PC 3). Just as the durational expression, the dynamic expression was accounted for by the components reflecting the structure of the piece. Even though the potential range of dynamic expressions is limited from *pp* to *p* in the score, pianists appeared to find a liberty of dynamic variations that could differentiate the ending section from the rest of the piece.

Moreover, the cross-sectional contrast between the final and the remaining sections, reflecting the global structure of the piece, appears to be the key in the pianist's expressions differentiating the two recordings. Pianists tend to execute the structural contrast in the range of dynamic expressions toward a more “averaged” manner in the audience-present recording, and this tendency is also the same as their durational expressions. Thus, pianists carefully control their individualistic expressions in live recordings. This supports that the artistically appealing performance holds the appropriate level of expressivity, which lies in between the mechanical and the exaggerated representations of the score while maintaining the structural contrasts of the piece (Shoda and Adachi, 2010, 2012).

## Study 3

Pianists in the present study executed the cross-sectional contrast of durational and dynamic expressions in a more “averaged” manner in the audience-present recording. How do such pianists' controls explain the differences in the audience's impressions between the audience-present and the audience-absent recordings? In Study 3, we explored the “causal” relationships between the pianist's context-bound expression of duration/dynamics and the listeners' responses through a multi-group path analysis (e.g., Ho, 1996). By doing so, we propose a model explaining the mechanism of social facilitation in piano performance.

### Method

A path analysis is a statistical method to describe the directed dependencies among a set of observed variables (Ho, 1996), which enables us to reveal interrelationships among independent and dependent variables. In this analysis, we tested “theoretically possible” models among listeners' ratings for the quality and their emotionally moving experiences, and two principal components reflecting the global structure of the piece (i.e., PC 2 for the smoothed beat duration, PC 3 for the smoothed dynamic range), all of which generated statistically significant differences between the recording conditions in Studies 1 and 2. For each of the listener's responses, the difference of the mean ratings for each performer from the audience-present to the audience-absent conditions was calculated for the path analysis. For each of the component scores of the performer's expression, we computed the difference of the absolute values from the audience-present to the audience-absent recordings, which represents the pianist's inclination toward “averageness” in the audience-present recording as compared with that in the audience-absent recording, as shown in Study 2. In this analysis, listeners were also categorized into three groups (i.e., least, moderately, most experienced listeners) in line with Study 1, so that we could identify “the common relationships among the groups” and “the different relationships as a function of the group” by a multi-group analysis (e.g., Ho, 1996).

**Figure 9:**
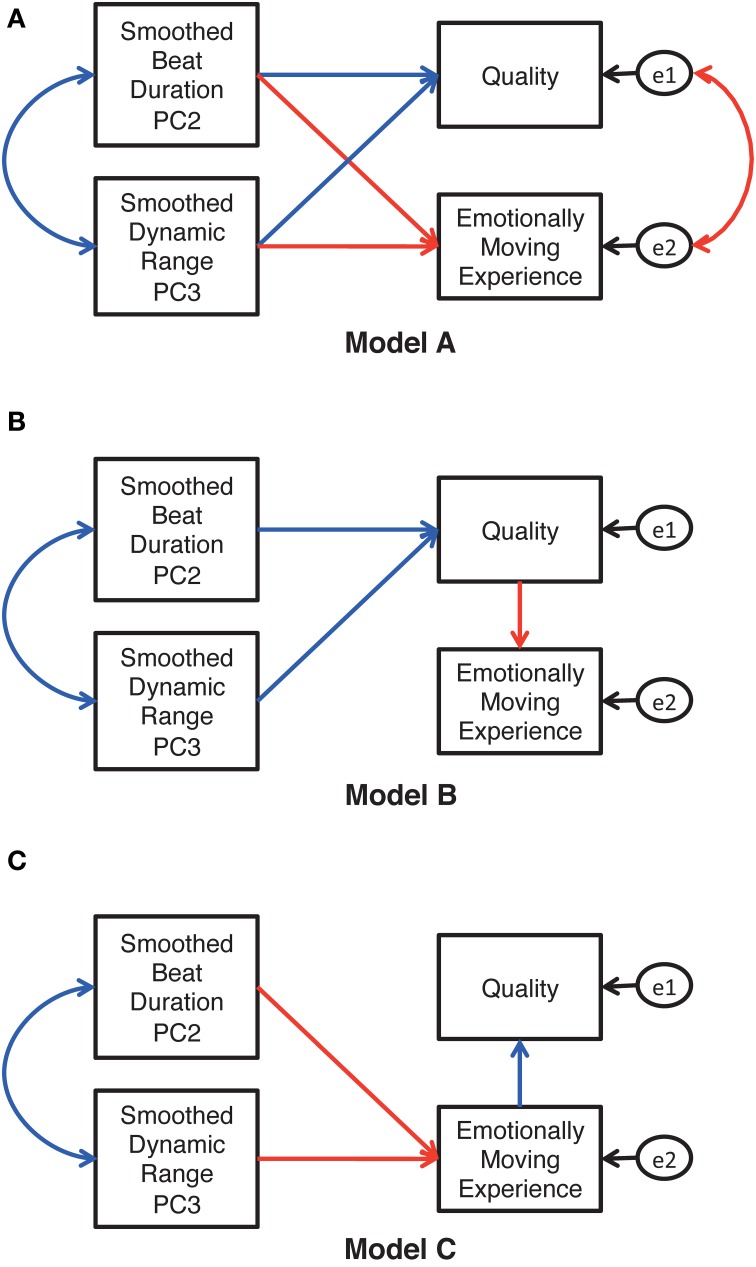
**Theoretically possible models for the relationship between the performer's expressions and the listener's responses**. The blue paths and correlations (bilateral arrows) indicate the common relationships regardless of the level of listeners' musical training, while the red paths and correlations indicate the relationships differing as a function of the level of musical training. The variables e1 and e2 are error variables.

Figure 9 shows three theoretically possible models. Based on the significant interaction between the recording condition and the training in Study 1, we predicted paths indicating “the common relationships regardless of training” (marked with blue lines) and those indicating “the different relationships as a function of training” (marked with red lines). In model A (Figure 9A), both listeners' responses (i.e., quality, emotionally moving experience) are predicted by both principal components. In Model B (Figure 9B), pianists' expressions determine listeners' judgment of the quality, which subsequently predicts their emotionally moving experience. Model C (Figure 9C) is a reversed model of model B. We tested these models by using the generalized least square method, whose degrees of fitness to the data were compared by the Akaike's Information Criteria (AIC; Akaike, 1973). The generalized least square method is appropriate for small samples such as ours (*N* = 13), for which the assumption of multivariate normal distribution could not be hypothesized (Kano and Miura, 2002). From the chosen model, we refined the model so as to fit the current data by removing insignificant paths.

### Results and discussion

**Figure 10:**
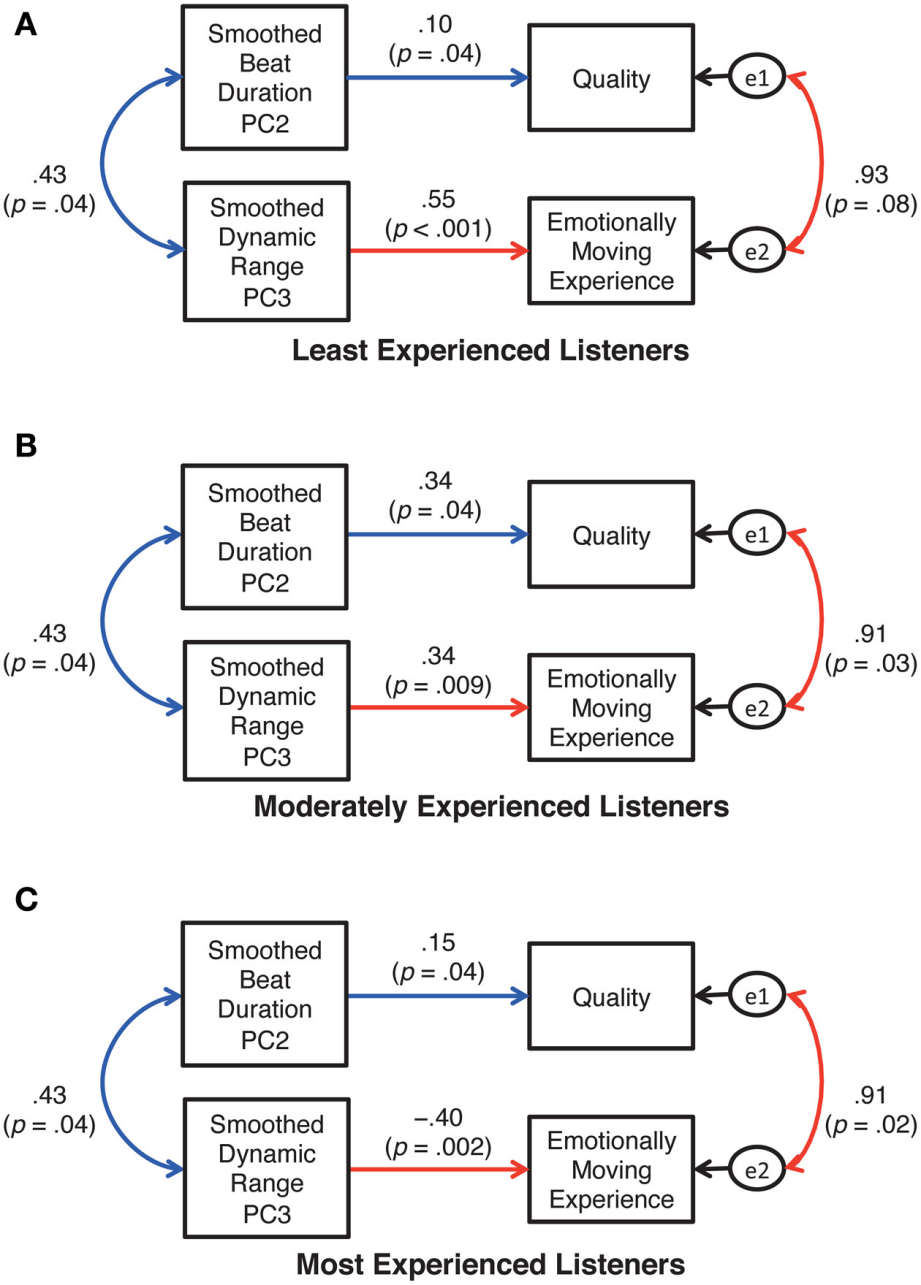
**The final model of the relationship between the performer's expressions and the listener's responses**. The variables e1 and e2 are error variables.

The AICs generated by three path analyses were 43.18, 49.37, and 50.45 for models A, B, and C, respectively. Thus, model A (Figure 9A) was the best of the three. By removing the insignificant paths from model A, the refined model (Figure 10) was confirmed to be the best fit for the current data with the criteria of goodness-of-fit indices, χ^2^ (14, *N* = 13) = 5.94, *p* = 0.97; GFI = 0.92, AGFI = 0.82, RMSEA = 0.001. Note that the coefficients described in Figure 10 were standardized, so that the values can be different across the training levels even for the paths specifying “the common relationship regardless of training (i.e., blue lines in Figure 10).” According to Figure 10, the durational and the dynamic parameters correlated positively with each other, indicating that pianists' inclinations toward “averaged” expressions are common between the parameters. This is in line with previous studies showing co-occurrence of performers' expressions for different expressive elements (e.g., Todd, 1992; Repp, 1996; Gingras et al., 2013).

However, these durational and the dynamic expressions influenced the listeners differently. The beat duration determined the listeners' evaluations of the quality regardless of the listeners' musical training. More specifically, the pianists' tendency toward an “averaged” temporal expression of the cross-sectional contrast between the final and the remaining sections in the live context elicited better evaluations in the listeners. This supports Repp (1997), in which the computer-generated “average” version sounded more appealing to listeners than did the majority of individual performances. The smoothed dynamic range, on the other hand, influenced the listeners' emotionally moving experience differently between those who had at least 16 years of musical training and those who had less musical training, as evident in the positive vs. negative effects of the corresponding paths. The most experienced listeners were moved more when the smoothed dynamic range between the final and the remaining sections was less averaged (Figure 10C), whereas the effect was reversed for the other listeners (Figures 10A,B). In other words, in the live recording, musically least or moderately experienced listeners' quality judgment and emotionally moving experiences were determined consistently by “averageness” of the pianist's cross-sectional expressions of temporal and dynamic variations. In the case of musically well-experienced listeners, their quality judgment was based on “averageness” in the cross-sectional temporal variations while their emotionally moving experiences were determined by less averaged (or more individualized) dynamic contrasts between the final and the remaining sections of the piece.

## General discussion

In the present study, we explored the mechanism of social facilitation through an advanced time-series analysis for pianists' acoustical expressions and the modeling statistics revealing the causal relationship between performers and listeners. First, we showed that the presence of the audience enhances the quality of the performance of *Träumerei*, although the effect sizes were not so large (η^2^_p_s = 0.04, and 0.03 for the quality and the emotionally moving experience, respectively). According to Zajonc (1965), the mere presence of others enhances the performer's arousal, which increases the likelihood of an organism to do well-learned responses better (“drive theory”). Although audiences in music performance are not merely present but actively seeking artistic experiences, showing spontaneous responses, and often being critical, “evaluation apprehension” (i.e., how the performer perceives the audience's response) does not influence the outcome of the performance in general, as indicated in the meta-analyses of 24,000 people by Bond and Titus (1983). The present results have reconfirmed this, and the social facilitation in piano performance may be explained by the drive theory. It should be noted, however, that the small effect sizes of the presence of the audience (η^2^_p_s = 0.04, and 0.03 for the quality and the emotionally moving experience, respectively) may imply large individual differences among listeners in evaluating performances through an auditory medium, since the pianist's body movement appears to determine the audience's evaluation of the quality and the emotionally moving experiences more directly than do the acoustical manipulations of tempo or dynamics (Shoda and Adachi, 2014). Lacking an access to the visual cues may also explain why the listeners' evaluations were not different as a function of performer in Study 1.

Second, we revealed three principal components of pianists' durational and dynamic expressions throughout the piece. The overall quantity, the contrast between the final and the remaining sections, and the control of the overall variability were identified as the principal components for both expressions, explaining over 70% of variance in each expression with each component contributing at least 11%. The first two components of the durational expressions were replications of Almansa and Delicado (2009), and they can be regarded as the global tempo and the structure-dependent temporal variation, respectively. The present study has shown that similar functions exist in pianists' dynamic expressions as well, and has added one more as a common function between durational and dynamic expressions. Replicability of these components needs to be tested with other pieces.

Nonetheless, the present study has indicated that pianists modulate the contrast between the final and the remaining sections both in durational and dynamic expressions in a more “averaged” manner in the live-recording situation. Why do pianists reduce their individuality in a live context? There are two possible answers. First, pianists tend to avoid risks of performing in an individualistic manner in front of an audience, who tend to prefer averaged expressions (Repp, 1997). Alternatively, the present finding may be culture-bound: Pianists in the present study avoided extreme expressions in the live recording context because of the general tendency of Japanese to display a neutral emotional demeanor. For example, Japanese subdue their emotional expressions, and even the vocal intonations tend to become monotonic in public (Jackson and Thurgate, 2011). The validity of these possible explanations needs to be tested through a cross-cultural study.

The present study has also indicated that the degree of “averageness” in the cross-sectional variations can explain the audience's higher evaluation of the performance quality and their emotionally moving experience, at least for listeners with a minimal or a moderate level of musical experience. Considering that performers' expressions of the structural contrasts are the principal rules in an artistic performance (e.g., Repp, 1990, 1992, 1995, 1996; Penel and Drake, 2004; Shoda and Adachi, 2012), it is not surprising that pianists differentiated their two recordings in relation to the structure-dependent (i.e., PC2 for the beat duration, PC3 for the dynamic range), rather than the beat-by-beat (i.e., PC1 for both parameters), expressions. Still remarkable is that the structural contrasts expressed by pianists could function as cues for the quality judgment and the emotionally moving experience of listeners, even though the components are the secondary (beat duration) and the tertiary (dynamic range) sources of their expressions in accordance with our functional principal components analyses. Thus, the present study has revealed that not only musicians (e.g., Repp, 1997; Yoshie et al., 2009a) but also non-music majors can identify the “subtle” differences between live and non-live recordings, evaluate their qualities, and reflect upon emotionally moving experiences accordingly, at least in the case of a familiar piece for them.

Finally, the multi-group path analysis revealed different determinants for the audience's quality judgment and their emotionally moving experiences across the two types of recordings. This appears to support that the quality of music and the listeners' emotional experiences do not necessarily overlap (e.g., Gabrielsson, 2001-2002). In particular, differences in the audience's quality judgment between the audience-present and the audience-absent recordings depended on “averageness” of the pianist's cross-sectional temporal variations regardless of musical experiences. Tempo is one of the most prominent cues in eliciting and interpreting emotional qualities of music, not only among musicians (e.g., Gabrielsson and Juslin, 1996; Juslin, 1997) but also among non-musicians and children (Adachi and Trehub, 1998; Adachi et al., 2004). The present finding appears to add another aspect of temporal expressions shared among listeners with different musical backgrounds.

Unlike the temporal expressions, the pianist's cross-sectional dynamic variations differentiated the listeners' emotionally moving experience between the two recordings as a function of musical training. Musically well-experienced listeners felt emotionally moved more with less averaged, or more individualized, dynamic expressions in the live recording. Considering that an emotionally moving experience is related to violation and fulfillment of the listener's expectancy, already embedded in the musical structure of a masterpiece (Meyer, 1956), the pianist's averaged expressions may be enough to induce emotional experiences in musically less experienced listeners. Well-experienced listeners, on the other hand, may have much more familiarity with the piece, indeed, they may even have played *Träumerei* on their own and have known that a Romantic piece should be performed with individualized expressions (Repp, 1995). This knowledge may have elicited a different level of expectancy in well-experienced listeners, requiring more unique dynamic expressions for their expectancy to be violated.

In the present study, we empirically verified the mechanism of why “the live recording sounds better and moves the listener more emotionally” by examining the interaction among the recording context, the acoustical expressions, the listener's judgments, and the listener's level of musical training. The acoustical expression in the final section of the piece is the key for both pianists and listeners in differentiating between the audience-present and the audience-absent recordings. The other factors, such as piece, cultural background, and the listener's familiarity and knowledge for the piece, should be explored in a future study.

### Conflict of interest statement

The authors declare that the research was conducted in the absence of any commercial or financial relationships that could be construed as a potential conflict of interest.

## Supplementary material

Supplementary Figure S1 **Three principal components functions for the smoothed beat duration generated by the functional principal components analysis**. The letters within the graph indicate section IDs.

Supplementary Figure S2 **Three principal components functions for the smoothed dynamic range generated by the functional principal components analysis**. The letters within the graph indicate section IDs.
